# Solving the Portfolio Optimization Problem on a Photonic Quantum Computer

**DOI:** 10.3390/e28070717

**Published:** 2026-06-23

**Authors:** Łukasz Grodzki, Mateusz Slysz, Grzegorz Waligóra

**Affiliations:** 1 Institute of Computing Science, Poznań University of Technology, Piotrowo 2, 61-138 Poznań, Poland; lukasz.grodzki@student.put.poznan.pl (Ł.G.); mateusz.slysz@doctorate.put.poznan.pl (M.S.); 2 Poznan Supercomputing and Networking Center, Institute of Bioorganic Chemistry, Polish Academy of Sciences, 61-139 Poznań, Poland

**Keywords:** quantum computing, photonic quantum computer, boson sampling, Binary Bosonic Solver, portfolio optimization, combinatorial optimization

## Abstract

Quantum computing offers new possibilities for solving combinatorial optimization problems with rapidly growing search spaces. Among emerging hardware platforms, photonic quantum computers based on boson sampling provide a promising approach for sampling-based optimization methods. In this work, we investigate the application of the Binary Bosonic Solver, a hybrid quantum–classical algorithm designed for photonic quantum processors, to the binary portfolio optimization problem derived from the classical mean–variance framework. In addition to evaluating the feasibility of solving such problems on photonic quantum hardware, we analyze the behavior of the Binary Bosonic Solver algorithm under different architectural and optimization parameters, including interferometer loop configurations and gradient estimation methods. Benchmark instances are generated using historical financial market data, and experiments are performed both on a photonic quantum computer simulator and on the ORCA PT-1 photonic quantum processor installed at Poznan Supercomputing and Networking Center, with results compared to those obtained using a classical optimization algorithm. The results demonstrate that portfolio optimization can be successfully executed on current photonic quantum hardware and that the Binary Bosonic Solver algorithm consistently produces feasible and high-quality solutions, highlighting the practical potential of photonic quantum computing for combinatorial optimization problems.

## 1. Introduction

Quantum computing has emerged as a promising paradigm for tackling complex optimization problems whose search spaces grow exponentially with the number of variables [1]. Quantum approaches aim to exploit quantum mechanical phenomena, including superposition and entanglement, to explore large solution spaces more efficiently than classical algorithms.

Many combinatorial optimization problems can be mapped to Quadratic Unconstrained Binary Optimization (QUBO) or Ising formulations, enabling their implementation within quantum frameworks [2]. Several quantum computing paradigms have been proposed for optimization tasks. One of the earliest is quantum annealing, where the solution to an optimization problem is encoded in the ground state of a quantum Hamiltonian and obtained through an adiabatic evolution of the system [3,4]. Another important class of methods consists of hybrid variational algorithms designed for Noisy Intermediate-Scale Quantum (NISQ) devices, such as the Quantum Approximate Optimization Algorithm (QAOA) [5], which iteratively optimizes parameterized quantum circuits using a classical optimizer. These quantum approaches have been explored for a variety of real-world applications. For instance, they have been applied in finance [6,7,8], logistics planning [9] and scheduling [10,11].

Beyond gate-based and annealing approaches, sampling-based quantum methods have also attracted attention as a way of generating candidate solutions to optimization problems. In these approaches, the quantum device produces samples from probability distributions that may emphasize high-quality solutions. Such methods are particularly relevant for hardware platforms where sampling from complex quantum distributions can be performed efficiently. Among the most promising platforms for implementing these ideas are photonic quantum computers, which exploit multi-photon interference in linear optical networks to generate highly nontrivial probability distributions. A prominent computational model implemented on photonic processors is boson sampling, first proposed by Aaronson and Arkhipov [12]. In boson sampling, multiple indistinguishable photons propagate through a linear interferometer, and the probability distribution of the output photon configurations is determined by matrix permanents, which are computationally hard to calculate classically.

Photonic quantum computing differs substantially from gate-based (NISQ) and quantum annealing architectures in both implementation and constraints. Gate-based platforms offer general-purpose programmability but operate at cryogenic temperatures, and their fixed qubit connectivity graphs can complicate circuit mapping as problem size grows. Quantum annealing devices are tailored to Ising-type optimization problems but rely on sparse connectivity, introducing embedding overhead that can scale quickly with instance size. Photonic systems encode information in optical modes and operate through interference-based sampling. Therefore, they can be implemented at room temperature without cryogenic requirements, which simplifies experimental deployment compared to superconducting architectures. As a result, photonic platforms are particularly compatible with standard research infrastructures, enabling their use in hybrid experimental setups alongside classical computing resources.

Although boson sampling was originally introduced as a model for demonstrating quantum computational advantage rather than universal quantum computation, its ability to generate complex probability distributions has motivated research into practical applications. Early work has explored this idea in the context of sampling-based optimization and learning tasks on photonic platforms [13,14], suggesting that structured optical interference may be exploited beyond purely demonstrational quantum advantage settings. Within this line of research, hybrid approaches have emerged that combine photonic sampling devices with classical feedback loops, aiming to bias the sampling distribution toward regions of higher objective value.

One such method is the Binary Bosonic Solver (BBS), a hybrid variational algorithm designed for photonic quantum processors [15]. In BBS, a boson sampling-type photonic circuit produces samples that represent candidate solutions for a binary optimization problem. Each sample corresponds to a possible assignment of binary variables, and the circuit’s controllable parameters, like beam-splitter angles in the interferometer, are updated iteratively using a classical optimizer to favor better solutions. By repeatedly sampling and updating the circuit, BBS explores the solution space efficiently, using the probabilistic nature of photonic interference to find high-quality solutions that are hard to obtain with classical methods. Subsequent work has explored its implementation within hybrid quantum–classical workflows and demonstrated its potential for solving combinatorial optimization problems using photonic quantum hardware [16,17].

Portfolio optimization is a fundamental problem in quantitative finance, where the objective is to allocate capital among a set of assets in order to achieve an optimal balance between expected return and financial risk. The modern formulation of this problem originates from the work of Harry Markowitz, who introduced the mean–variance framework that remains the cornerstone of modern portfolio theory [18]. In this formulation, investors seek to maximize expected return while minimizing the variance of portfolio returns, leading to a constrained optimization problem over asset allocations.

While the classical mean–variance portfolio optimization problem can be solved efficiently using convex quadratic programming techniques, practical portfolio construction often involves additional constraints such as cardinality limits, minimum investment sizes, and transaction costs [19]. These constraints introduce discrete decision variables that transform the problem into a mixed-integer quadratic program [20]. As a result, the optimization problem becomes combinatorial in nature, with the number of possible portfolios growing exponentially with the number of assets [21].

In this paper, we consider a binary formulation of the portfolio optimization problem, where each asset is either included or excluded from the portfolio. This formulation represents a simplified computational model and should be viewed as a test case for combinatorial optimization rather than a fully realistic portfolio construction framework. The problem can be efficiently addressed using the BBS algorithm, and we evaluate instances on the ORCA PT-1 photonic quantum processor installed at Poznańskie Centrum Superkomputerowo-Sieciowe (Poznan Supercomputing and Networking Center, PSNC) as well as on classical simulators of such a device. The results are compared with classical optimization methods, including Simulated Annealing, to assess the performance of BBS on combinatorial financial optimization problems.

The main novelty of this work lies in applying an existing algorithm—the Binary Bosonic Solver (BBS)—to a financially relevant binary portfolio optimization problem. To the best of our knowledge, this is the first study investigating portfolio optimization within the BBS framework on both simulated and physical photonic quantum hardware. The proposed setup does not introduce a new benchmarking methodology or algorithmic advancement; instead, it provides a representative evaluation of the algorithm under different operating conditions, including variations in photonic architecture, optimization parameters, and comparison with a classical Simulated Annealing baseline, in order to assess the practical applicability of photonic quantum optimization methods on current and future-generation photonic hardware.

The remainder of this paper is organized as follows. Section 2 describes the materials and methods used in this study, including the formulation of the portfolio optimization problem and the experimental setup used to run the BBS algorithm. Section 3 presents the experimental results and the comparison with classical optimization approaches. Section 4 discusses the obtained results and their implications, and Section 5 concludes the paper.

## 2. Materials and Methods

### 2.1. Binary Bosonic Solver

The first version of the BBS algorithm was proposed in [22] and later extended in [15]. The algorithm utilizes samples generated by a photonic quantum computer performing a boson sampling task as candidate solutions for combinatorial optimization problems. Each output sample of the photonic circuit is interpreted as a binary vector in the solution space, whose quality is assessed using a classical objective function. Based on this evaluation, the interferometer parameters are updated using a classical optimizer, thereby increasing the probability of generating higher-quality solutions in subsequent iterations.

The use of boson sampling circuits is particularly advantageous in this setting, as multi-photon interference in linear optical networks induces highly structured probability distributions that are difficult to simulate classically. By tuning the interferometer parameters, the sampling distribution can be continuously reshaped, allowing the algorithm to preferentially explore regions of the solution space associated with improved objective values.

The behavior and performance of the BBS algorithm are influenced by several hyperparameters that control both the optimization loop and the sampling process performed by the photonic circuit.

One important parameter is the number of optimization iterations, which determines how many times the algorithm performs the sampling–evaluation–update cycle. Increasing the number of iterations allows the optimizer to gradually adapt the circuit parameters and improve the probability of generating high-quality candidate solutions, although it also increases the overall computational cost.

Another key parameter is the number of samples generated in each iteration. Since the algorithm relies on stochastic samples from the photonic circuit, using a larger number of samples provides a more reliable estimate of the objective function landscape and reduces the influence of statistical fluctuations. However, this also increases the runtime of each iteration.

Different strategies for sample postselection can also be employed when evaluating the candidate solutions generated during each iteration. Since the photonic circuit produces a set of stochastic samples, multiple approaches can be used to aggregate this information when guiding the optimization process. For example, the objective value may be computed using the average score of all sampled solutions, the best solution observed in a given batch, or the most frequently occurring sample. Each strategy leads to slightly different optimization dynamics.

The size of the optimization problem is defined by the number of binary decision variables. In photonic implementations this size is related to the number of available optical modes in the quantum processor, which limits the dimensionality of the solution vectors that can be generated directly by the boson sampling circuit. To address this limitation, the BBS algorithm can employ a technique known as tiling. In this approach, multiple binary vectors produced by the boson sampling circuit are concatenated to form a longer solution vector representing a larger optimization instance. This allows the algorithm to be applied to problems whose dimensionality exceeds the native size supported by the photonic hardware.

Another factor influencing the performance of the algorithm is the optimization method used to update the circuit parameters. In principle, gradient-based optimization methods are commonly used in variational quantum algorithms. However, computing exact gradients using classical backpropagation or full stochastic gradient descent is often impractical in sampling-based quantum settings due to the high cost of circuit evaluations and shot noise. As a result, practical implementations frequently rely on efficient gradient estimation techniques tailored to quantum hardware. Among these, the parameter-shift rule provides an important special case in which gradients can be evaluated analytically by exploiting the structure of parameterized quantum gates [23,24]. This property arises directly from quantum interference effects and the unitary evolution of parameterized gates, making the method fundamentally quantum in origin rather than a numerical approximation in the classical sense. In contrast, stochastic methods such as Simultaneous Perturbation Stochastic Approximation (SPSA) [25] estimate gradients using randomized finite differences, typically requiring only two circuit evaluations per iteration regardless of parameter dimension. While this makes SPSA highly scalable, it introduces additional variance compared to the exact (but more structure-dependent) parameter-shift rule. Gradient-free optimizers, such as COBYLA (Constrained Optimization BY Linear Approximation) [26], can also be employed. These methods avoid gradient estimation entirely; however, they are generally less efficient for high-dimensional variational quantum circuits and may exhibit slower convergence compared to gradient-informed approaches.

The structure of the interferometer also has a major impact on algorithm performance. While changing the number of modes seems to not be an option, since it should be the same as the problem size, one can expand the grid horizontally by simply adding more layers of beam-splitters. This allows the algorithm to solve more complex problems due to the increased number of tunable parameters, which significantly enhances the expressive power of the optical circuit.

The connectivity between modes also plays a significant role in the sample generation process. Interferometers with a greater number of connections between different modes may offer greater potential for quantum entanglement of optical paths. A special case of an interferometer used in this study is the Time Bin Interferometer (TBI) [27], which works just the same as the diffraction grid except it operates in the time domain rather than the spatial domain. In this architecture, instead of using multiple parallel physical paths for the modes, photons are routed through an optical medium that can be thought of as delay loops. The interference occurs between photons separated by specific time intervals, effectively translating the spatial complexity of a physical grid into a sequence of temporal delays, which drastically reduces the physical footprint required to scale the system. One can manipulate the lengths of the loops to determine which modes are coupled together by the beam splitters, effectively changing the connectivity between modes.

Finally, the input photonic state used in the boson sampling circuit also affects the sampling behavior of the algorithm. The input state determines how photons are distributed across the input modes of the interferometer before propagation through the optical network. Increasing the number of photons generally leads to richer interference patterns and more complex output distributions. However, generating multi-photon states is experimentally challenging due to photonic hardware limitations [28], which often motivates the use of relatively sparse input configurations, such as alternating patterns of occupied and empty modes. To compensate for this limited diversity in the physical input states, the algorithm can employ additional trainable classical parameters, such as the probabilities of bit flips applied to sampled solutions, which broaden the effective search space explored during the optimization process.

### 2.2. Portfolio Optimization Problem Formulation

#### 2.2.1. Continuous Portfolio Optimization

Continuous portfolio optimization aims to allocate wealth among *n* assets in a way that balances expected return and risk. Let $w={\left(w_{1},w_{2},\ldots ,w_{n}\right)}^{⊤}$ denote the vector of portfolio weights, where $w_{i}$ is the fraction of total wealth invested in asset *i*. According to [18], the expected return of the portfolio is

(1) $$\mu_{p}=w^{⊤}\mu ,$$

and the portfolio variance (a measure of risk) is

(2) $$\sigma_{p}^{2}=w^{⊤}\Sigma w,$$

where μ is the vector of expected returns and Σ is the covariance matrix of asset returns.

It is important to note that stock investment risk differs from regular risk. In investing, assuming diversification, the probability of total loss is incredibly low and intractable. This is why volatility is the primary measure of risk typically expressed as standard deviation or variance. To reduce this risk, one can increase the number of stocks. However, this approach can be optimized by taking into account the covariance of the assets. If the chosen stocks had low covariance in the past, there is a high chance that while some of them decline, others rise, thus reducing the overall variance of the portfolio, which can be calculated from covariance matrix like in Equation (2).

The optimization problem can be formulated in two equivalent ways. First, as a return-maximization problem for a given level of risk:

(3) $$\begin{array}{cccc} \; & \mathrm{maximize} & \; & \mu_{p}=w^{⊤}\mu \\ \; & \mathrm{subject}\;\mathrm{to} & \; & \sigma_{p}^{2}=w^{⊤}\Sigma w,\\ \; & \; & & \sum_{i=1}^{n}w_{i}=1,\\ \; & \; & & w_{i}\geq 0,\;i=1,\ldots ,n. \end{array}$$

Alternatively, one can minimize risk for a given target return $\mu^{*}$:

(4) $$\begin{array}{cccc} \; & \mathrm{minimize} & \; & \sigma_{p}^{2}=w^{⊤}\Sigma w\\ \; & \mathrm{subject}\;\mathrm{to} & \; & w^{⊤}\mu =\mu^{*},\\ \; & \; & & \sum_{i=1}^{n}w_{i}=1,\\ \; & \; & & w_{i}\geq 0,\;i=1,\ldots ,n. \end{array}$$

To solve this constrained optimization problem, one can use the method of Lagrange multipliers (see, e.g., [29]).

In a continuous setup, one can generate infinitely many portfolios by sampling random vectors of weights w that satisfy the constraints. Similarly, both the return-maximization and risk-minimization optimization problems can be solved for infinitely many target returns or risk levels.

Doing so allows one to trace out the efficient frontier, also known as the Pareto front, which represents the set of portfolios that achieve the best possible trade-off between expected return and risk. Portfolios on this frontier are Pareto optimal, meaning that no other portfolio exists that offers a higher expected return for the same level of risk, or equivalently, a lower risk for the same expected return.

In practice, the efficient frontier is typically generated computationally because the continuous weight space is high-dimensional and analytical solutions exist only under certain simplifying assumptions. Two common computational approaches are:

- Random sampling of portfolios: One generates a large number of random weight vectors w satisfying the constraints (e.g., $\sum_{i}w_{i}=1$ and $w_{i}\geq 0$). For each sampled portfolio, the expected return $\mu_{p}=w^{⊤}\mu$ and risk $\sigma_{p}^{2}=w^{⊤}\Sigma w$ are computed. Plotting these points produces an approximate scatter of feasible portfolios, with the upper-left boundary corresponding to the efficient frontier.
- Optimization for multiple targets: Another approach is to solve the risk-minimization problem repeatedly for a range of target returns $\mu^{*}$ (or equivalently, solve the return-maximization problem for a range of risk levels). Each solution yields a point $\left(\sigma_{p},\mu_{p}\right)$ on the efficient frontier. This method produces a smooth and precise curve, which is usually preferred for visualization and analysis.

**Figure 1:**
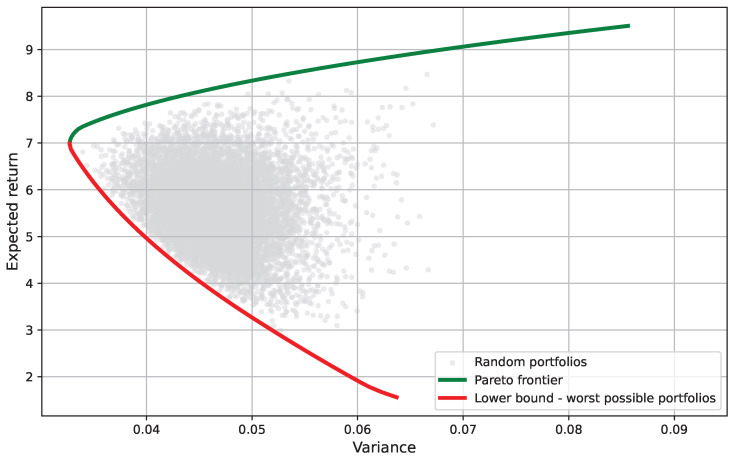
Example of a Pareto frontier for a 5-asset portfolio with continuous allocation. Gray dots represent portfolios with randomly generated weight vectors. The green line denotes the Pareto frontier, calculated by minimizing the risk for a given expected return using the Least Squares Programming algorithm for 50 equidistant points. The red line represents the worst possible portfolios, calculated in the same manner. Although these lines are drawn from a discrete set of points, the continuous nature of the weight vectors’ values ensures they closely approximate the actual analytical boundaries.

These computational methods are important because the weight space grows exponentially with the number of assets, making exhaustive enumeration impossible. We used them on an example problem instance to efficiently approximate the continuous Pareto front and produce the risk-return plot used in practice, visualized in Figure 1.

#### 2.2.2. Binary Portfolio Optimization

In binary portfolio optimization, the allocation decision for each asset is restricted to a discrete choice, typically 0 or 1, representing whether an asset is excluded or included in the portfolio. Let $x_{i}\in \left\{0,1\right\}$ denote the inclusion variable for asset *i*. The binary portfolio problem can be formulated as

(5) $$\begin{array}{cccc} \; & \mathrm{maximize} & \; & \mu_{p}=\sum_{i=1}^{n}\mu_{i}x_{i}\\ \; & \mathrm{subject}\;\mathrm{to} & \; & \sigma_{p}^{2}=\sum_{i=1}^{n}\sum_{j=1}^{n}x_{i}x_{j}\sigma_{ij}\leq \sigma^{*},\\ \; & \; & & \sum_{i=1}^{n}x_{i}=b,\\ \; & \; & & x_{i}\in \left\{0,1\right\},\;i=1,\ldots ,n, \end{array}$$

where $\mu_{i}$ is the expected return of asset *i*, $\sigma_{ij}$ is the covariance between assets *i* and *j*, $\sigma^{*}$ is the target portfolio risk, and *b* is the number of assets to include.

Binary portfolio optimization is a combinatorial problem, which is generally NP-hard, but despite the computational challenges, binary optimization is important for practical scenarios where fractional investments are impossible or where investment rules impose discrete constraints [18].

In binary portfolio optimization, the set of feasible portfolios is discrete and finite, but grows combinatorially with the number of assets. Therefore, the efficient frontier is typically approximated rather than derived analytically. The common computational approaches are:

- Exhaustive enumeration: For small numbers of assets, one can evaluate all possible combinations of $x_{i}\in \left\{0,1\right\}$ that satisfy the constraints. Each combination yields a portfolio $\left(\sigma_{p},\mu_{p}\right)$, and the Pareto-optimal portfolios form the discrete efficient frontier. This method guarantees finding the true optimal frontier but is feasible only for small *n* due to combinatorial explosion.
- Heuristic or metaheuristic search: For larger asset sets, exact enumeration is computationally infeasible. Different heuristics are used to explore the space of feasible portfolios. Each candidate portfolio is evaluated for return and risk, and the best-performing candidates collectively approximate the efficient frontier.

**Figure 2:**
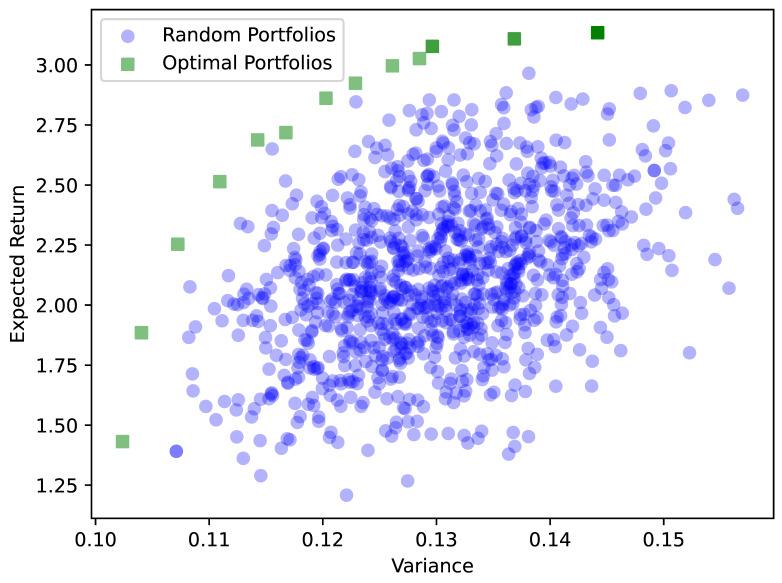
Discrete Pareto frontier for problem size of 20. Blue dots represent randomly generated portfolios. Green squares represent portfolios with the highest expected return for a given variance. The random portfolios form a dense cloud due to statistical effects. In the multidimensional space of possible asset allocations, there are more weight combinations that result in highly diversified, average market exposures than combinations leading to extreme, optimal boundaries. Consequently, randomly generated weight vectors naturally cluster in the middle, far from the Pareto frontier.

Although the resulting frontier is discrete and may contain gaps, it still provides investors with a practical guide to selecting portfolios that achieve a good trade-off between expected return and risk under binary constraints. An example of such a discrete frontier is shown on Figure 2.

### 2.3. Experimental Setup

#### 2.3.1. Portfolio Optimization Instances

The data set consists of instances generated using historical market data from Yahoo Finance public API, obtained via the yfinance (version 1.2.0) [30] Python (version 3.12.10) library. The generation process for each n∈{5,10,15,20,25} followed a structured pipeline.

For each value of *n*, we randomly selected five distinct sets of *n* tickers from S&P500 index. For each set, we retrieved historical adjusted closing prices covering the last five years. This data served as the basis for calculating average daily returns and covariance matrix of the assets. All of these sets are expected to be highly distinct, as their elements are sampled from a set with a cardinality exceeding 500.

To create a diverse range of problem instances, we utilized a full-space search to identify the global minimum and maximum possible variance for each asset set, that did not break any of the constraints. We then defined 21 equidistant variance targets within this range.

The first point (minimum variance) was excluded from the final set because there is only one unique feasible solution with the target variance equal to the minimum. Repetition of this process across various ticker groups allowed us to generate 100 unique covariance matrices and expected return vectors for each *n*, resulting in 500 instances in total.

The 100 generated instances for each *n* exhibit significant diversity regarding the problem’s energy landscape. For each *n*, five separate sets were selected, inherently creating distinct landscape groups. Each of these groups is further partitioned by selecting different target volatilities, which further alters the problem landscape.

#### 2.3.2. Experiments on the Photonic Quantum Computer Simulator

To evaluate the performance of BBS, we tested it across various binary portfolio optimization instances and compared it against a classical algorithm with similar characteristics. We selected Simulated Annealing (SA) [31], implemented via the simanneal [32] Python library, due to its probabilistic nature and the ability to evaluate solutions using the exact same objective functions as BBS. This choice enables a direct comparison between two stochastic optimization approaches under comparable objective-function evaluation budgets. We note that SA was used as a representative classical baseline rather than a state-of-the-art portfolio optimization method, and the goal of the comparison is to assess the behavior of BBS rather than establish competitiveness with the strongest available classical approaches.

One of the parameters of the Simulated Annealing is the number of iterations. Although the BBS algorithm utilizes a parameter with the same name, simply equating these values would yield an unfair comparison. Performance should instead be evaluated based on the total number of objective function evaluations. This approach is necessary because the algorithm is ultimately intended for execution on specialized, continuously evolving hardware, meaning the ultimate speed of individual operations remains unknown. In SA, the number of function evaluations corresponds directly to the number of iterations. In contrast, for BBS, this relationship is dictated by the chosen gradient calculation method.

Additional parameters of SA include the temperature bounds $T_{\max}$ and $T_{\min}$. $T_{\max}$ represents the initial temperature value, which is reduced in each subsequent iteration until it reaches $T_{\min}$, according to the following formula:

(6) $$T_{k}=T_{\max}\cdot {\left(\frac{T_{\min}}{T_{\max}}\right)}^{\frac{k}{I}}$$

where $T_{k}$ is the temperature at iteration *k* and *I* is the total number of iterations.

The default values for $T_{max}$ and $T_{min}$ in the simanneal library are 25,000 and 2.5, respectively. These values were retained for our experiments, as preliminary testing indicated that modifying them did not yield performance improvements.

For this set of experiments, we employed the parameter-shift method, which is the most precise one for computing gradients, in exchange for speed. This approach requires $n_{e}$ evaluations of the objective function:

(7) $$n_{e}=I\times S\times \left(\sum_{j=1}^{K}\left(m-l_{j}\right)+2m+1\right),$$

where *I* is the number of iterations, *S* is the number of samples drawn per iteration, *m* is the number of modes and *l* is the list of the loop lengths. To guarantee a fair comparison, the SA algorithm was constrained to match the exact number of objective function evaluations necessary for BBS across all experiments.

To assess the performance of the algorithms, we tracked two primary metrics: the percentage of optimal solutions found and the average relative solution quality. The relative quality was determined by first identifying the global best and worst feasible solutions—defined as those strictly satisfying all constraints—through a full-space search. Having established these absolute bounds, the average percentage of the optimal solution was calculated using the following equation:

(8) $$C_{rel}=\frac{C_{alg}-C_{opt}}{C_{max}-C_{opt}}\times 100\%,$$

where $C_{alg}$ is the cost returned by the algorithm, $C_{opt}$ is the optimal cost (the lowest) and $C_{max}$ is the highest cost from all feasible solutions.

This equation corresponds to the normalization of the feasible solutions’ cost range. Consequently, while this normalization limits the practical interpretation of portfolio behavior to strict hierarchical rankings in real-world scenarios, it provides a robust framework from an algorithmic perspective. In terms of cost function values, it clearly demonstrates the solution’s proximity to the optimum, remaining entirely independent of the varying optimal and worst-case values across different instances.

The experimental setup utilized a simulated interferometer comprising *m* modes, where *m* strictly corresponds to the problem size, and incorporated two different configurations of loops of lengths [1, 3] (2 loops) and [1, 3, 9] (3 loops). These power-law configurations facilitate relatively unrestricted photon propagation through the interferometer, as longer loops enable connections between more distant modes. Such configurations are frequently employed by the hardware manufacturer and exhibit a high probability of forward compatibility with future hardware architectures. An alternating sequence of zeros and ones was employed as the initial input state. The alternating occupation of input modes (filling every second mode) provides a compromise between the experimental cost of generating high-quality multi-photon input states and sufficient coverage of the solution space. This choice is consistent with current photonic hardware limitations, where increasing photon number and state complexity significantly reduces state preparation success rates [33]. The learning rates were configured to 0.01 for the beam-splitter angles and 0.05 for the bit-flip probabilities. These specific learning rates were selected because they demonstrated robust performance in previous experiments across various problem domains. The objective of this research was not to tune hyperparameter configurations, but rather to benchmark the algorithm’s typical behavior. The algorithm processed each problem instance over 200 iterations, drawing 50 samples per iteration. Furthermore, while implementing tiling within the simulator is technically feasible and could yield reduced simulation times, it typically incurs a penalty in overall solution quality. To ensure maximum accuracy and establish a rigorous baseline for evaluation, tiling was intentionally omitted from all simulator-based experiments. The omission of tiling in simulation experiments is related to differences between simulated and physical photonic hardware. In current devices, the limited number of available optical modes requires tiling to embed larger optimization instances into the hardware architecture. However, in future-generation photonic hardware with increased mode capacity, tiling may be significantly reduced or eliminated, making the simulation setting more representative of such architectures.

Additionally, we compared more various configurations of loop lengths—[1], [1, 2], [1, 2, 4], and [1, 2, 4, 8]—using the SPSA method for gradient calculation. This shift in methodology was motivated by SPSA’s position at the opposite end of the accuracy–cost trade-off relative to the parameter-shift rule. Specifically, SPSA sacrifices strict precision in favor of computational efficiency, requiring substantially fewer evaluations of the objective function $n_{e}=I\times S\times 2\times q$, where *q* is any integer greater than 0. Typically *q* is equal to 1 and so it was in our experiments. For every loop length configuration we solved 100 instances of size 20—a subset of instances used in previous experiments.

#### 2.3.3. Experiments on the Photonic Quantum Computer

Physical hardware experiments were executed on the ORCA PT-1 device hosted at PSNC. The PT-1 system’s interferometer features two loops with fixed lengths of one, both of which were utilized in our setup. The hardware provides eight modes, which dictated the specific sizes of our problem instances. Additionally we compare the results of two-loop system with the one using only one loop.

To accelerate computation on physical hardware, the number of BBS iterations was reduced to 100, with 30 samples drawn per iteration. Although utilizing SPSA could further decrease the computation time, it substantially increases the risk of noise-driven gradient estimations compared to the parameter-shift method. To ensure the accuracy and stability of the optimization process, we therefore elected to use the parameter-shift rule. The learning rates remained identical to those used in the simulated experiments.

For these specialized hardware tests, we generated an additional set of instances using a framework analogous to the simulator experiments. For each problem size n∈{6, 12, 18, 24}, we randomly selected one set of *n* assets from the S&P 500 index. We conducted a full-space search to identify the global minimum and maximum variances among feasible solutions. From this range, we defined 11 equidistant variance targets, yielding 10 valid problem instances per value of *n*.

We selected values of *n* to be multiples of six. This choice was driven by the hardware’s operational limitations: utilizing six modes requires using three photons, assuming an alternating input state of zeros and ones. The calculation time on the physical device is highly dependent on the total number of photons, *p*. Because single-photon generation is a probabilistic process [33], executing a *p*-photon experiment requires *p* consecutive successful generations, causing the overall execution time to scale exponentially with *p*. We determined that operating in a three-photon regime provides an optimal practical trade-off: it avoids prohibitively long execution times while ensuring sufficient non-trivial multi-photon interference to actively leverage the quantum characteristics of the device.

Furthermore, using multiples of six naturally enables a straightforward application of the tiling technique. Because of that, we can simply use *t* tiles, where $t=\frac{n}{6}$.

## 3. Results

### 3.1. Simulation Results

**Figure 3:**
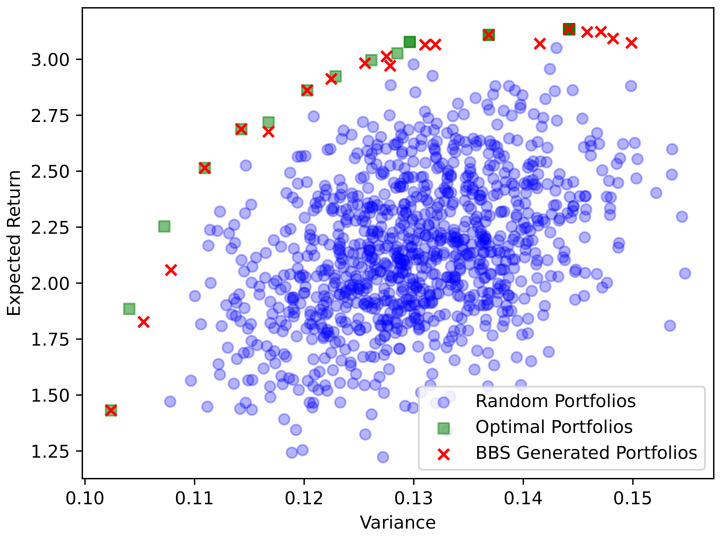
Simulation results for problem size of 20 with the use of two loops across 21 different volatility targets. Blue circles represent randomly generated portfolios. Green squares represent discrete Pareto frontier. Red crosses represent portfolios created from the BBS solutions.

In this section, we present the results of our simulation experiments. The BBS algorithm found feasible solutions across all 500 benchmark instances and constructed discrete Pareto frontiers for all smaller instances. In order to create such a frontier, one would have to know the optimal solutions for every target volatility of a given set of assets. It is natural that for larger problem sizes, probabilistic algorithms do not always find the optima, and here it was also the case. Figure 3 provides a visual representation of the discrete Pareto frontier in relation to the portfolios obtained using BBS for one of the asset sets. We present our previously chosen metrics in Table 1 for the two-loop experiment and in Table 2 for the three-loop experiment.

**Figure 4:**
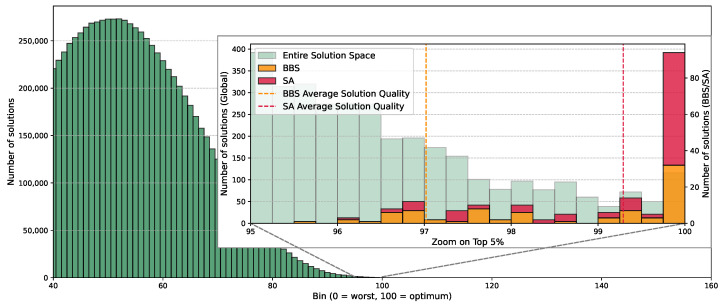
Distribution of relative solution quality across all 100 problem instances of size 20, magnified to highlight solutions whose cost is no more than 5% away from the optimum. The green histogram represents the underlying distribution of all feasible solutions. The orange and crimson histograms depict the distribution of solutions generated by BBS and SA, respectively. Additionally, dashed lines indicate the average solution quality achieved by each algorithm.

To precisely quantify the number of solutions within specific intervals of relative quality, we generated histograms, presented in Figure 4, detailing the relative solution quality distribution across all feasible solutions, with a specifically magnified view of the high-quality tail.

To further investigate the influence of the underlying architecture, we evaluated the impact of incorporating a third loop into the simulated interferometer. In this expanded setup, the device comprised three loops of lengths one, three, and nine, following the power law. For these specific tests, problem instances of size five had to be excluded because the problem dimensionality must be strictly greater than or equal to the maximum loop length. If the problem size were smaller than the largest loop, photons would either simply escape the interferometer in the spatial representation or be detected outside the experiment’s time bounds in the temporal representation.

Table 3 illustrates how the performance metrics are impacted by varying the number of loops within the interferometer. For this configuration, we employed the SPSA method for gradient estimation to verify that the algorithm continues to converge on feasible solutions. Because increasing the number of loops inherently expands the parameter space of the model, this setup favors the use of SPSA, which offers significant computational speedups over alternative gradient calculation methods in high-dimensional spaces in exchange for accuracy.

### 3.2. Physical Hardware Results

In this experimental setup, we compare the algorithm’s performance using one-loop versus two-loop configurations across various problem sizes. A direct comparison between the BBS executed on physical hardware and SA was omitted due to the current high levels of hardware noise. Such comparisons are deferred until the hardware becomes sufficiently fault-tolerant, ensuring that physical noise does not significantly interfere with the algorithm’s operation. For identical reasons, the parameter-shift rule was employed for the gradient calculation in this experiment. Combining the inherently stochastic nature of SPSA with existing hardware noise would likely yield inconclusive results. In Table 4, we present the results of this experiment.

## 4. Discussion

The experimental results demonstrate that the BBS algorithm consistently identifies feasible solutions across a diverse range of portfolio optimization instances of varying dimensions, evaluated under multiple algorithmic configurations. Furthermore, when operating under the primary setup detailed in this study, the algorithm successfully located the global optimum in over 74% of the instances.

Comparing BBS (using the parameter-shift method for gradient calculation) with SA, BBS returned slightly worse results. While both algorithms found global optima for smaller problem sizes, the difference in average solution quality grew slowly as the problem size increased. For a problem size of n=25, the difference in the average quality metric was around two percentage points.

This discrepancy corresponds to an approximate 2% variance in actual portfolio returns. Relative to the entire solution space, this difference is marginal, as illustrated in Figure 4. Because these relative solution quality metrics lie within the extreme tail of the distribution, the density of solutions between these two thresholds is significantly lower than it would be closer to the median. Specifically, out of 10,607,634 total feasible solutions evaluated for instances of size 20, only 639 solutions ranked within the top 2%. Furthermore, 533 solutions fell between the 2% and 4% thresholds, and 948 solutions between the 4% and 6% thresholds, demonstrating a highly sparse distribution of optimal and near-optimal solutions.

BBS identified fewer global optima compared to SA. However, the combined success rates show that BBS and SA find optimal solutions for distinct subsets of problem instances. This difference in performance may be related to their different algorithmic mechanisms: BBS operates as a gradient-based optimization approach, whereas SA relies on random state changes.

In the experiment evaluating the impact of incorporating an additional loop into the interferometer, BBS demonstrated improved performance. This suggests that expanding the parameter space can improve performance under the tested configurations.

The final simulation experiment provided particularly noteworthy results. A comparison of various loop length configurations, utilizing SPSA for gradient estimation, demonstrated that increasing the interferometer’s parameter count does not strictly correlate with enhanced algorithmic performance in this setup. Although configurations with the highest number of parameters yielded the best average solutions, the performance difference compared to setups with the minimum parameter count was negligible. Furthermore, while a restricted number of objective function evaluations substantially degraded the quality of SA’s solutions, BBS exhibited significantly greater resilience, experiencing only a small decline in solution quality despite requiring 38.5 times fewer objective function evaluations. Notably, both algorithms consistently returned exclusively feasible solutions. Although BBS found more global optima, in this setup, it is more appropriately viewed as an efficient algorithm for quickly discovering high-quality feasible solutions rather than a guaranteed global optimizer.

The results from the physical hardware experiments demonstrate that the BBS algorithm is viable in early-stage development devices, successfully identifying global optima for problem sizes up to n=24. Across both tested loop configurations, the algorithm consistently returned feasible solutions for every instance. Furthermore, incorporating an additional loop generally increased the average solution quality and enabled BBS to discover a greater number of global optima. The only exception occurred with instances of size 12, where the higher-parameter configuration identified fewer optimal solutions, which was most likely caused by the probabilistic nature of the algorithm.

Due to the current state of the photonic hardware, the scope of feasible experiments remains highly restricted. The limited number of available configurations, constrained problem sizes, and hardware noise all contribute to these limitations. Consequently, current research investigating the behavior of algorithms designed for this architecture must be predominantly simulator-based, while executions on actual physical hardware currently serve primarily as proofs of feasibility, initial benchmarks, and preliminary experiments that facilitate ongoing hardware development.

## 5. Conclusions

We have performed experimental evaluations of the Binary Bosonic Solver (BBS) on multiple instances of the binary portfolio optimization problem, using both classical simulations and photonic quantum hardware. Based on these experiments, we conclude that BBS is a promising algorithm capable of solving chosen optimization problems, including the binary portfolio optimization problem. It can be utilized to search for global optima but is often most useful for finding high-quality feasible solutions, when operating under limited objective function evaluation budgets.

The comparison of the BBS algorithm with Simulated Annealing demonstrates that, despite the absence of a consistent performance advantage over the classical baseline, certain BBS hyperparameter setups achieved better solution quality, while maintaining relatively low computational cost. Hence, the results indicate that careful hyperparameter selection is critical for achieving competitive performance of BBS under the considered experimental conditions.

Higher overall solution quality was achieved using the parameter-shift rule for gradient calculation. This performance is primarily attributed to the extensive budget allocated for cost function evaluations, enabling the algorithm to achieve results comparable to, or marginally worse than SA. Conversely, SPSA demonstrated a distinct advantage in rapid convergence in relation to lower budget of cost function evaluations. In scenarios where quick generation of high-quality solutions is prioritized, the SPSA-driven approach proved superior, effectively outperforming SA. Furthermore, an underlying trend indicates that the parameter-shift rule scales better with complex circuit architectures. In contrast, under the experimental settings considered in this work, SPSA appeared less capable of effectively exploiting an increased number of trainable parameters.

Despite these promising results, several limitations should be acknowledged. The experiments were performed on relatively small problem instances, reflecting the current constraints of available photonic hardware rather than real-world portfolio sizes. In addition, the benchmark formulation is intentionally simplified and does not incorporate several practical financial considerations. The comparison with classical methods is also limited to Simulated Annealing, which provides a baseline of comparable stochastic structure but does not represent the full range of state-of-the-art optimization techniques. Finally, the study focuses on first-generation ORCA photonic devices, which are still subject to hardware-specific limitations. These constraints also influence certain experimental design choices, as the selection of parameters must remain compatible with the current hardware capabilities.

A promising direction for future research is to further improve and systematically explore extensions, hyperparameter configurations, and optimization strategies for the BBS algorithm, as well as benchmarking its performance against established classical algorithms across different optimization problems. In particular, a more comprehensive study of how different parameter settings and control strategies affect convergence behavior and solution quality could lead to improved performance. A promising avenue for future research is the development of a hybrid gradient estimation strategy. By alternating both gradient calculation methods used in this study within a single optimization run, the algorithm could utilize the parameter-shift method to accurately track the global gradient of the loss landscape, while deploying SPSA to quickly explore the immediate local solution space.

The future trajectory of both the BBS algorithm and its underlying hardware is rapidly evolving. Because simulating interferometers with more than 25 modes and multiple loops is computationally demanding, future experiments scaling to significantly larger problem sizes will necessitate the use of specialized, physical hardware. As the number of available physical modes and parameters in photonic quantum computers increases, it may become possible to address substantially larger and more complex optimization problems. This may enable further investigation of photonic quantum devices for solving larger-scale optimization problems and facilitate more comprehensive comparisons with advanced classical optimization methods.

## Figures and Tables

**Table 1:** Simulation results for two loops.

Problem Size	BBS Avg Quality [%]	SA Avg Quality [%]	Optima BBS [%]	Optima SA [%]	Combined Optima [%]
5	100.00	100.00	100.00	100.00	100.00
10	100.00	100.00	100.00	100.00	100.00
15	99.37	100.00	84.00	100.00	100.00
20	97.02	99.29	28.00	57.00	68.00
25	95.28	97.16	5.00	5.00	10.00

**Table 2:** Simulation results for three loops.

Problem Size	BBS Avg Quality [%]	SA Avg Quality [%]	Optima BBS [%]	Optima SA [%]	Combined Optima [%]
10	100.00	100.00	100.00	100.00	100.00
15	99.45	100.00	84.00	100.00	100.00
20	97.66	99.62	32.00	70.00	80.00
25	95.77	97.65	5.00	15.00	20.00

**Table 3:** Comparison of BBS and SA algorithm results with different loop lengths configurations.

Loop Lengths Configuration	Number of Loops	BBS Avg Quality [%]	SA Avg Quality [%]	BBS Optima [%]	SA Optima [%]
[1]	1	94.79	91.18	11.00	2.00
[1, 2]	2	94.77	91.82	14.00	0.00
[1, 2, 4]	3	94.67	92.23	10.00	3.00
[1, 2, 4, 8]	4	95.09	92.18	12.00	1.00

**Table 4:** Physical hardware experiments’ results with the use of one and two loops.

Problem Size	BBS Avg Quality [%]	BBS Avg Quality [%]	Optima BBS [%]	Optima BBS [%]
	1 Loop	2 Loops	1 Loop	2 Loops
6	100.00	100.00	100.00	100.00
12	100.00	99.98	100.00	90.00
18	94.54	97.02	10.00	20.00
24	92.28	93.13	0.00	10.00

## Data Availability

Data are contained within the article.

## Abbreviations

The following abbreviations are used in this manuscript:

BBS	Binary Bosonic Solver
COBYLA	Constrained Optimization BY Linear Approximation
NISQ	Noisy Intermediate-Scale Quantum
QAOA	Quantum Approximate Optimization Algorithm
QUBO	Quadratic Unconstrained Binary Optimization

PSNC	Poznan Supercomputing and Networking Center
SA	Simulated Annealing
SPSA	Simultaneous Perturbation Stochastic Approximation
TBI	Time Bin Interferometer

## References

[1] Volpe D., Orlandi G., Turvani G. (2025). Improving the solving of optimization problems: A comprehensive review of quantum approaches. Quantum Rep..

[2] Lucas A. (2014). Ising formulations of many NP problems. Front. Phys..

[3] Kadowaki T., Nishimori H. (1998). Quantum annealing in the transverse Ising model. Phys. Rev. E.

[4] Farhi E., Goldstone J., Gutmann S., Lapan J., Lundgren A., Preda D. (2001). A quantum adiabatic evolution algorithm applied to random instances of an NP-complete problem. Science.

[5] Farhi E., Goldstone J., Gutmann S. (2014). A quantum approximate optimization algorithm. arXiv.

[6] Orús R., Mugel S., Lizaso E. (2019). Quantum computing for finance: Overview and prospects. Rev. Phys..

[7] Egger D.J., Gambella C., Marecek J., McFaddin S., Mevissen M., Raymond R., Simonetto A., Woerner S., Yndurain E. (2020). Quantum computing for finance: State-of-the-art and future prospects. IEEE Trans. Quantum Eng..

[8] Herman D., Googin C., Liu X., Sun Y., Galda A., Safro I., Pistoia M., Alexeev Y. (2023). Quantum computing for finance. Nat. Rev. Phys..

[9] Liu P., Parkinson S., Best K. (2025). Quantum and Quantum-Inspired Optimisation in Transport and Logistics: A Systematic Review. Smart Cities.

[10] Kurowski K., Pecyna T., Slysz M., Różycki R., Waligóra G., Wȩglarz J. (2023). Application of quantum approximate optimization algorithm to job shop scheduling problem. Eur. J. Oper. Res..

[11] Fu K., Liu J., Chen M., Zhang H. (2025). Solving Flexible Job-Shop Scheduling Problems Based on Quantum Computing. Entropy.

[12] Aaronson S., Arkhipov A. (2011). The computational complexity of linear optics. Proceedings of the Forty-Third Annual ACM Symposium on Theory of Computing.

[13] Brod D.J., Galvão E.F., Crespi A., Osellame R., Spagnolo N., Sciarrino F. (2019). Photonic implementation of boson sampling: A review. Adv. Photonics.

[14] Arrazola J.M., Bromley T.R., Rebentrost P. (2018). Quantum approximate optimization with Gaussian boson sampling. Phys. Rev. A.

[15] Makarovskiy A., Slysz M., Grodzki Ł., Siera D., Farnsworth T., Clements W.R., Rydlichowski P., Kurowski K. (2025). A Binary Optimisation Algorithm for Near-Term Photonic Quantum Processors. arXiv.

[16] Slysz M., Kurowski K., Waligóra G. (2024). Solving Combinatorial Optimization Problems on a Photonic Quantum Computer. arXiv.

[17] Slysz M., Grodzki Ł., Rydlichowski P., Siera D., Kurowski K., Waligóra G., Węglarz J. (2026). Solving combinatorial optimization and machine learning problems on hybrid near-term quantum photonic computers. Future Gener. Comput. Syst..

[18] Markowitz H.M. (2008). Portfolio Selection: Efficient Diversification of Investments.

[19] Chang T.J., Meade N., Beasley J.E., Sharaiha Y.M. (2000). Heuristics for cardinality constrained portfolio optimisation. Comput. Oper. Res..

[20] Bienstock D. (1996). Computational study of a family of mixed-integer quadratic programming problems. Math. Program..

[21] Cornuejols G., Peña J., Tütüncü R. (2018). Optimization Methods in Finance.

[22] Bradler K., Wallner H. (2021). Certain properties and applications of shallow bosonic circuits. arXiv.

[23] Schuld M., Bergholm V., Gogolin C., Izaac J., Killoran N. (2019). Evaluating analytic gradients on quantum hardware. Phys. Rev. A.

[24] Facelli G., Roberts D.D., Wallner H., Makarovskiy A., Holmes Z., Clements W.R. (2024). Exact gradients for linear optics with single photons. arXiv.

[25] Spall J.C. (1992). Multivariate stochastic approximation using a simultaneous perturbation gradient approximation. IEEE Trans. Autom. Control.

[26] Powell M.J.D., Gomez S., Hennart J.P. (1994). A direct search optimization method that models the objective and constraint functions by linear interpolation. Advances in Optimization and Numerical Analysis.

[27] Motes K.R., Gilchrist A., Dowling J.P., Rohde P.P. (2014). Scalable boson sampling with time-bin encoding using a loop-based architecture. Phys. Rev. Lett..

[28] Eisaman M.D., Fan J., Migdall A., Polyakov S.V. (2011). Invited review article: Single-photon sources and detectors. Rev. Sci. Instrum..

[29] Nocedal J., Wright S.J. (2006). Numerical Optimization.

[30] Aroussi R. (2026). Yfinance. https://github.com/ranaroussi/yfinance.

[31] Kirkpatrick S., Gelatt C.D., Vecchi M.P. (1983). Optimization by simulated annealing. Science.

[32] Perry M. (2019). simanneal: Python Module for Simulated Annealing. https://github.com/perrygeo/simanneal.

[33] Forbes I., Ghafari F., Deacon E.C.R., Singh S.P., Lavie E., Yard P., Shaw R.D., Laing A., Tischler N. (2025). Heralded generation of entanglement with photons. Rep. Prog. Phys..

